# Supplementary material for: Sea level regulated tetrapod diversity dynamics through the Jurassic/Cretaceous interval
Source: Nat Commun. 2016 Sep 2;7:12737. doi: 10.1038/ncomms12737 (PMC5025807; doi:10.1038/ncomms12737)
Supplement: Supplementary Information — Supplementary Figures 1-7, Supplementary Tables 1-3, Supplementary Methods and Supplementary References [file ncomms12737-s1.pdf]

Supplementary Figures

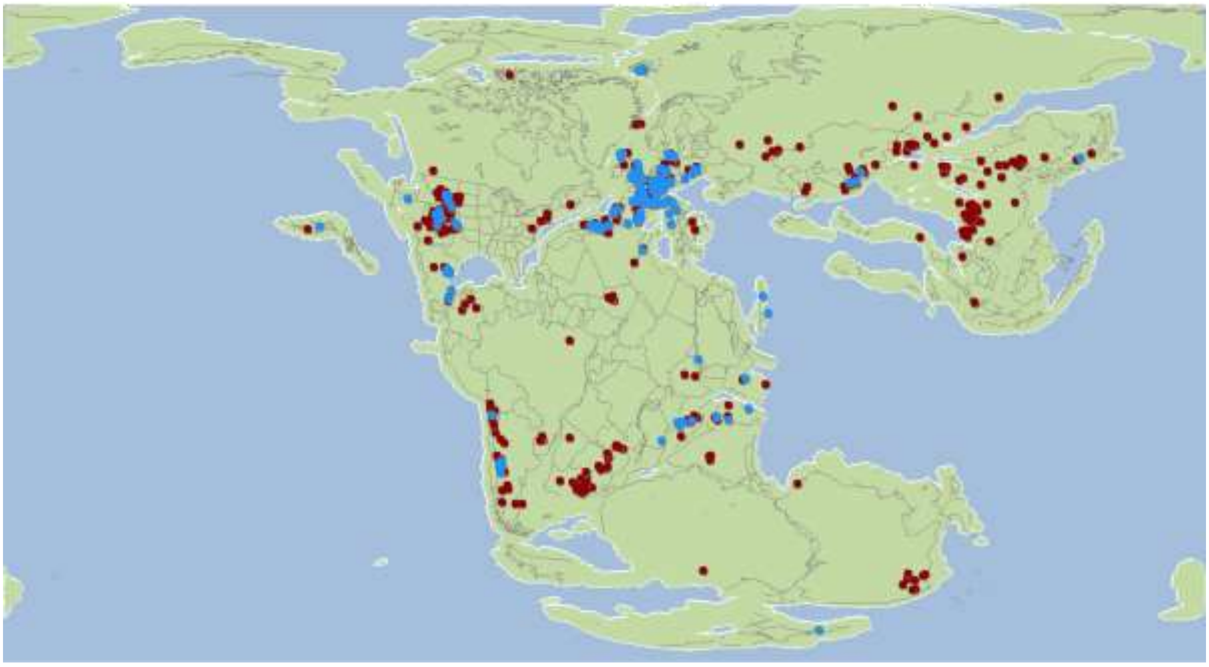

Supplementary Figure 1. Jurassic marine (blue) and non-marine (brown) tetrapod body fossil occurrences.

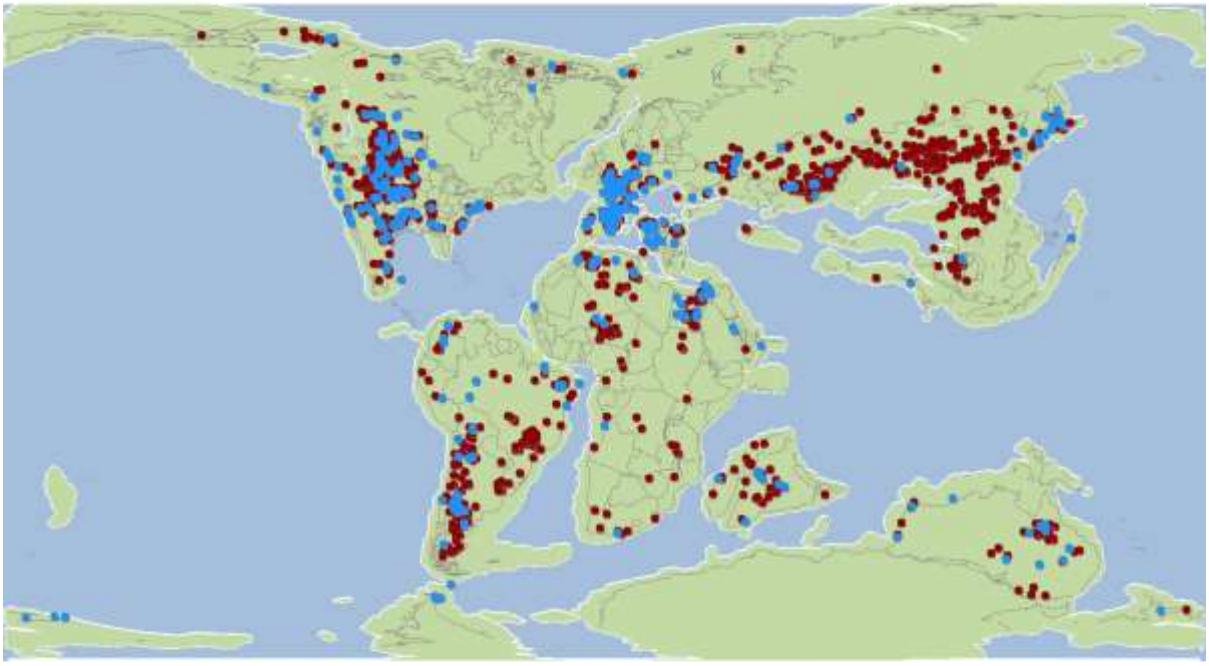

Supplementary Figure 2. Cretaceous marine (blue) and non-marine (brown) tetrapod body fossil occurrences.

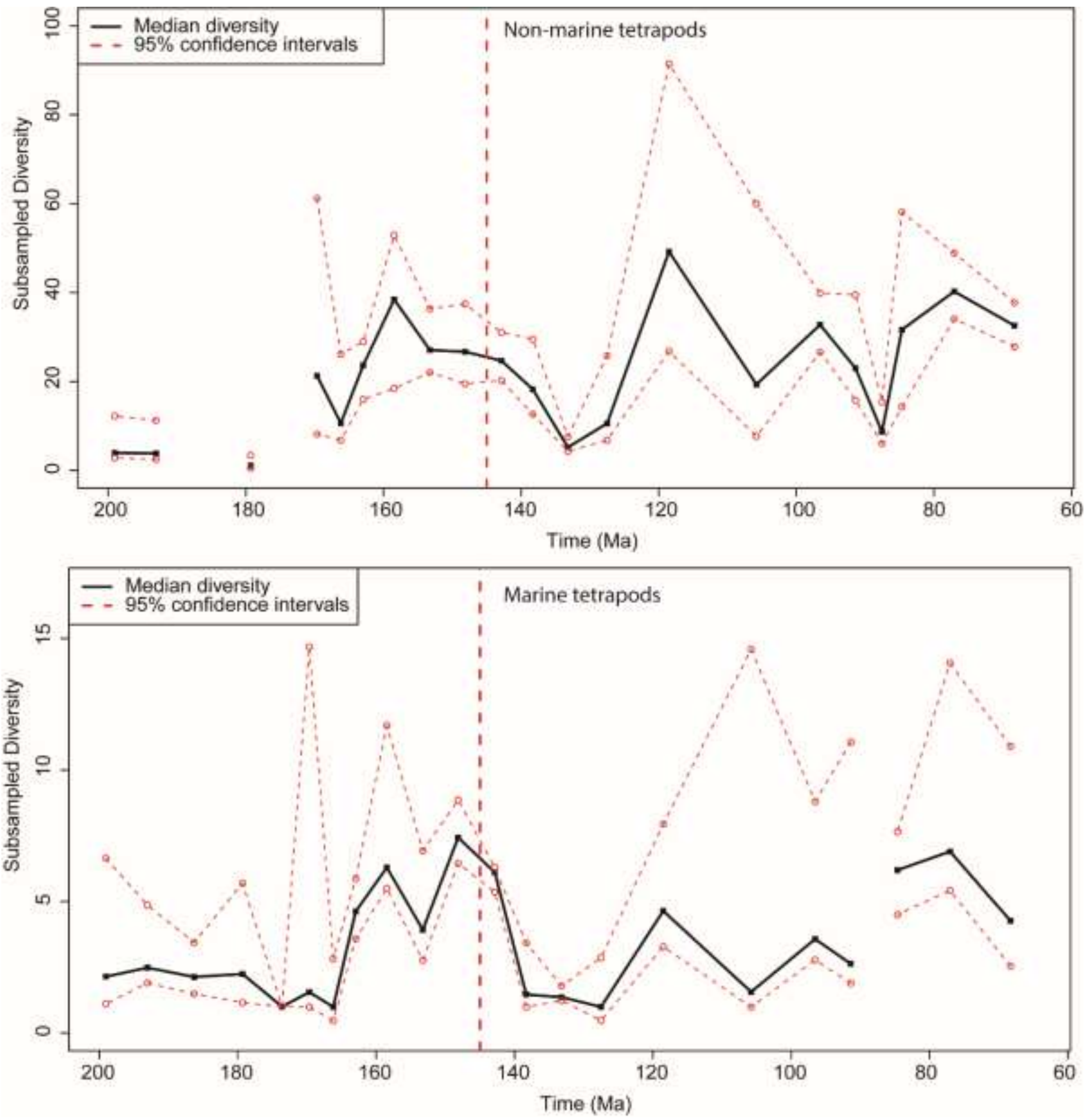

Supplementary Figure 3. Bootstrapped SQS diversity for non-marine and marine tetrapods.

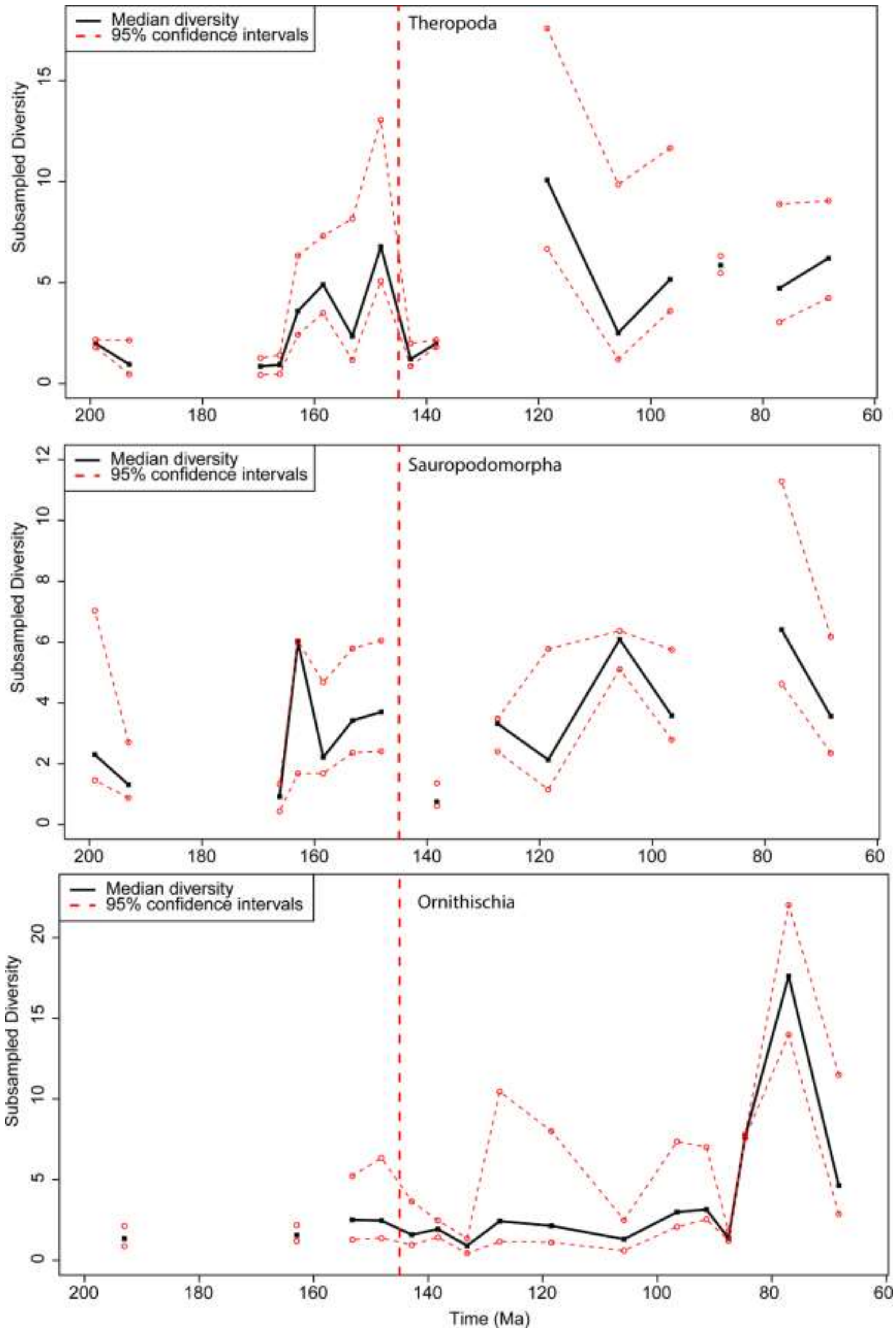

Supplementary Figure 4. Bootstrapped SQS diversity for the three major dinosaurian clades.

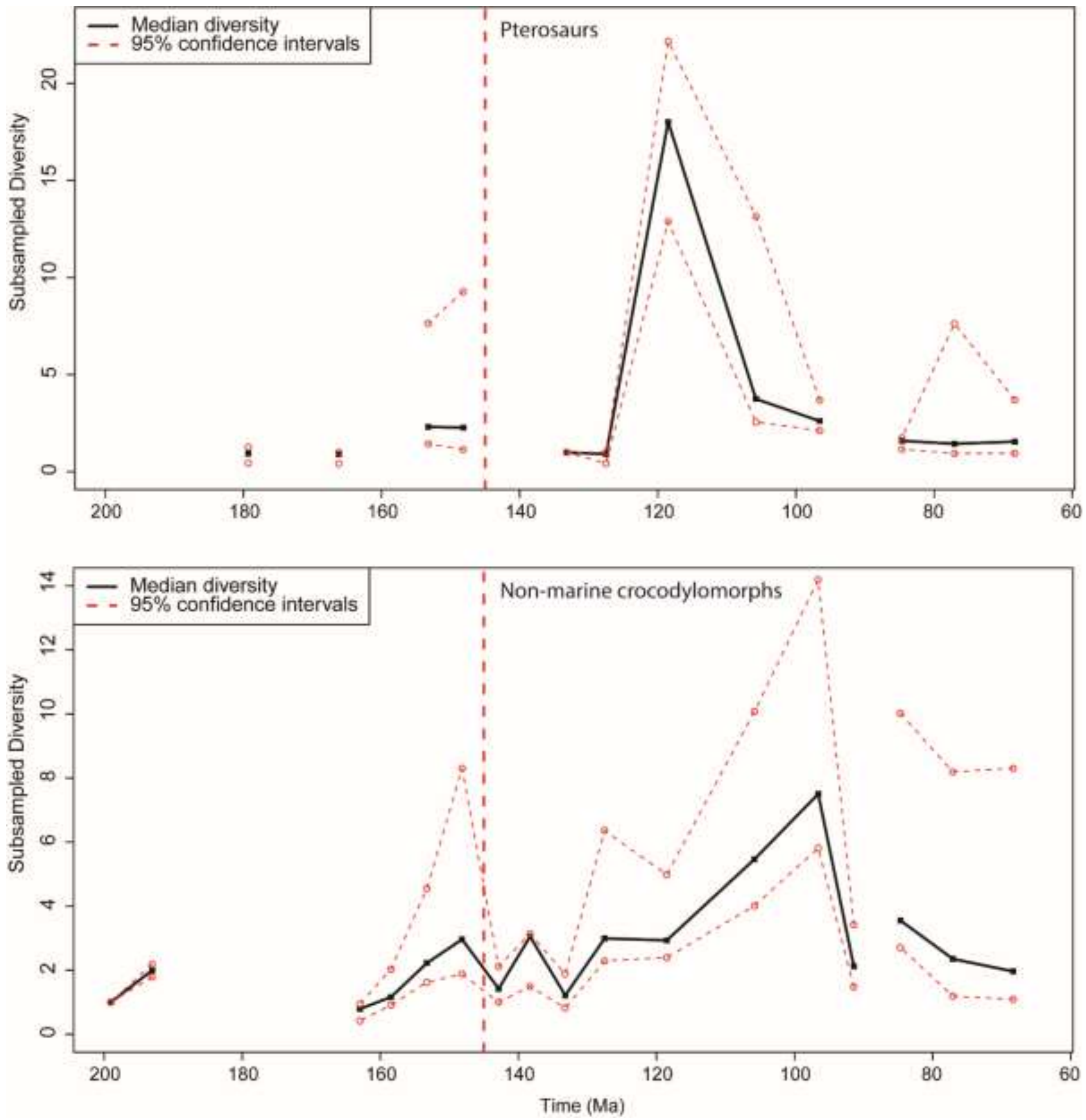

Supplementary Figure 5. Bootstrapped SQS diversity for pterosaurs and non-marine crocodylomorphs.

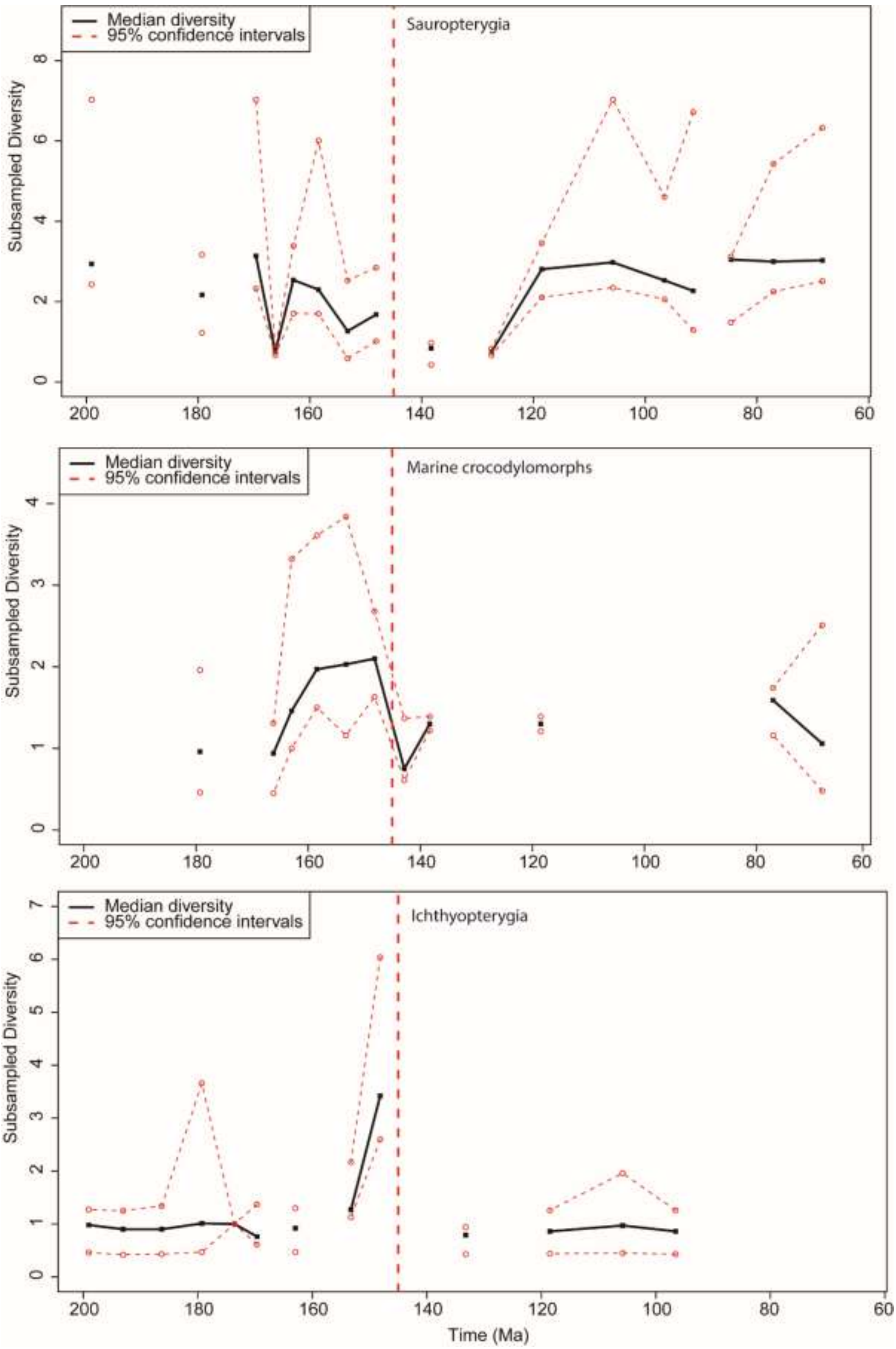

Supplementary Figure 6. Bootstrapped SQS diversity for marine tetrapod groups.

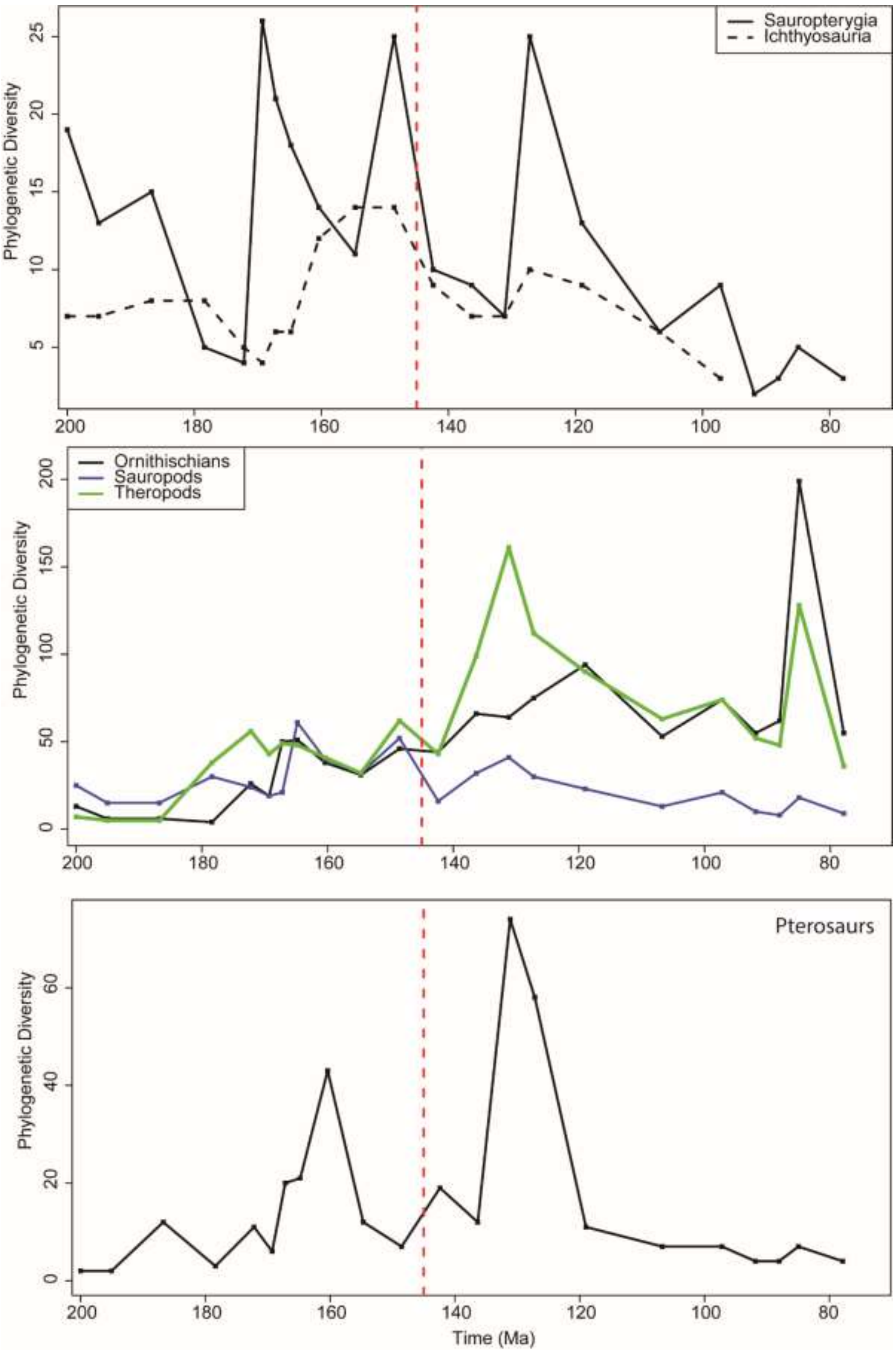

Supplementary Figure 7. Phylogenetic diversity estimates for different tetrapod clades.

Supplementary Tables

Supplementary Table 1. Details on our approximately equal 10 million year time bin scheme

Abbreviation	10 million year time bin	Stage equivalent	Temporal range
K8	Cretaceous 8	Maastrichtian	70.6–66.0
K7	Cretaceous 7	Campanian	84.9–70.6
K6	Cretaceous 6	Turonian–Santonian	94.3–84.9
K5	Cretaceous 5	Cenomanian	99.7–94.3
K4	Cretaceous 4	Albian	112.6–99.7
K3	Cretaceous 3	Aptian	125.5–112.6
K2	Cretaceous 2	Hauterivian–Barremian	136.4–125.5
K1	Cretaceous 1	Berriasian–Valanginian	145.5–136.4
J6	Jurassic 6	Kimmeridgian–Tithonian	155.7–145.5
J5	Jurassic 5	Callovian–Oxfordian	164.7–155.7
J4	Jurassic 4	Bajocian–Bathonian	171.6–164.7
J3	Jurassic 3	Toarcian–Aalenian	183–171.6
J2	Jurassic 2	Pliensbachian	189.6–183
J1	Jurassic 1	Hettangian–Sinemurian	201.6–189.6

Supplementary Table 2. Stage based time binning scheme

Abbreviation	Stage	Bin base (Ma)
K12	Maastrichtian	70.6
K11	Campanian	84.9
K10	Santonian	85.8
K9	Coniacian	89.3
K8	Turonian	94.3
K7	Cenomanian	99.7
K6	Albian	112.6
K5	Aptian	125.5
K4	Barremian	130
K3	Hauterivian	136.4
K2	Valanginian	140.2
K1	Berriasian	145.5
J11	Tithonian	150.8
J10	Kimmeridgian	155.7
J9	Oxfordian	161.2
J8	Callovian	164.7
J7	Bathonian	167.7
J6	Bajocian	171.6
J5	Aalenian	175.6
J4	Toarcian	183
J3	Pliensbachian	189.6
J2	Sinemurian	196.5
J1	Hettangian	201.6

Supplementary Table 3. Collections (colls), references (refs), raw genera (gen) and body fossil occurrences (occs) for each major taxonomic group analysed.

Taxon	Full data set		Stage				10 myr			
	Colls	Occs	Refs	Gen	Colls	Occs	Refs	Gen	Colls	Occs
Aves	211	314	53	55	64	105	134	130	159	250
Chelonioidea	95	109	31	21	48	56	53	27	75	85
Choristodera	226	232	53	5	163	167	83	11	211	216
Crocodyliformes (marine)	302	249	144	28	264	310	160	31	284	330
Crocodyliformes (non-marine)	807	823	187	73	474	484	316	131	667	681
Ichthyosauria	206	260	103	31	144	192	128	36	173	224
Lepidosauromorpha	594	614	89	93	365	381	159	153	545	564
Lissamphibia	561	631	101	67	468	522	135	78	537	600
Mammaliaformes	1908	2240	173	237	1420	1634	249	313	1791	2089
Ornithischia	1826	1896	409	160	841	868	733	275	1531	1590
Pterosauria	330	395	91	63	169	203	142	107	256	312
Sauropodomorpha	916	988	194	93	366	414	341	156	701	765
Sauropterygia	389	430	137	68	214	242	193	90	326	362
Testudines	875	1489	221	115	397	770	348	150	719	1266
Theropoda	1739	1806	327	166	919	964	600	266	1479	1540
**SUM**	**10985**	**12476**	**2313**	**1275**	**6316**	**7312**	**3774**	**1954**	**9454**	**10874**

Supplementary Methods

Age of the Jurassic/Cretaceous boundary. The lack of understanding regarding biotic and abiotic dynamics across the Jurassic/Cretaceous (J/K) boundary is emphasised by the fact that it is the only Mesozoic interval without a Global Boundary Stratotype Section (GBSS)[1]. Recently Vennari *et al.*[2] argued for a J/K boundary age of 140 million years old (Ma), based on a combination of biostratigraphic markers, isotopes, and sedimentation rates. Traditionally, most research has followed the proposed age of 144.6 ± 0.8 Ma for the J/K boundary, based on Mahoney *et al.*[3] and more recently Wimbledon *et al.*[4]. However, this date has yet to be formally recognised by the International Commission on Stratigraphy[1], and the age of the base of the Berriasian remains uncertain. For the purposes of the present study, we follow the absolute age of ~145.0 Ma proposed by Mahoney *et al.*[3], and utilised in the International Chronostratigraphic Chart[1].

Assembly of a global tetrapod occurrence dataset. For the current study, we selected tetrapods for several reasons. Firstly, a newly compiled Mesozoic dataset has been built within the *Paleobiology Database* (PaleoDB; http://www.paleobiodb.org/), representing one of the largest and most comprehensive datasets ever assembled for tetrapod groups[5] (Supplementary Data 1). Secondly, the impact of heterogeneous sampling on this group has been explored in a variety of ways for the different inclusive clades, suggesting that sampling biases have had a major impact on our reading of their 'raw' fossil record[6-16]. Thirdly, different tetrapod groups have varying spatiotemporal distributions, so offer a valuable perspective into heterogeneity of the fossil record. Finally, tetrapods occupied almost every possible environment or combination of environments throughout the Mesozoic era. This is reflected in their broad range of ecomorphologies, from small-bodied terrestrial animals to macropredaceous pelagic forms.

Our dataset spans the entirety of the Jurassic to Cretaceous (201–66 Ma), and is based on a newly compiled fossil occurrence dataset [5] (Supplementary Figures 1 and 2). This comprises a near-comprehensive record of tetrapods within the PaleoDB, accessed 31st May, 2015. Despite issues with supra-specific assessments of biodiversity patterns[17,18], we selected to use genera as genera and species diversity generally track each other for Mesozoic tetrapods[10,19], and this allows for the inclusion of specifically-indeterminate occurrences in the dataset (e.g., *Allosaurus* sp.), which would otherwise be excluded in a species-level analysis. Only body fossils are included, and we elect to include occurrences with qualifiers such as cf. and aff. recorded in the PaleoDB. The full dataset comprises 12,476 individual fossil occurrences from 10,985 collections. PaleoDB collections represent discrete fossil assemblages, divided based upon their stratigraphic range, collection history, lithology and scale.

Taxonomic categories are based on major clades that passed through the J/K boundary, or radiated in the Early Cretaceous (Supplementary Table 1). For example, we include Lepidosauromorpha, but exclude Mosasauroidea as a Late Cretaceous marine radiation. We also treat Choristodera as a separate group. We partition dinosaurs into four separate sub-groups due to

their proportionally large sample sizes: Sauropodomorpha, Ornithischia, Theropoda (excluding Aves) and Aves. For turtles, we use the more inclusive grouping of Testudines, but treat marine Chelonioidea as a separate Cretaceous sub-group. For Crocodyliformes, we follow Mannion *et al.*[10] and Tennant *et al.*[20] by dividing this into two sub-groups: the first, comprising non-marine forms including coastal, fluvial and semi-aquatic taxa, and a second comprising fully marine (pelagic) taxa, including Thalattosuchia, Gavialoidea, Dyrosauridae, and some pholidosaurids (Supplementary Data 1).

Each taxonomic sub-group was further geographically assigned to approximately contiguous palaeocontinental regions: Africa, Asia, Europe, South America, and North America. For each regional dataset, the record of fossil occurrences is patchy and discontinuous. Such patchiness is especially prevalent in Gondwanan regions, in which sampling occurs in distinct spatiotemporal clusters[8]. The full list of countries included in each regional data subset is given in Supplementary Data 1. The few tetrapod fossils from regions such as Australia, Antarctica, and Indo-Madagascar were included in global analyses, but were too sparse to warrant analysis on a regional level.

Time binning scheme. The geological timescale used here is based upon the Standard European Stages and absolute dates provided by Gradstein *et al.*[21]. Many recent studies of tetrapod diversity have used geological stages[6-8], or grouped stages into approximately equal length intervals[10,12,22,23] or finer sub-divisions[19,24,25], to pool taxonomic occurrences. Here, occurrence data for each group were pooled into: (1) approximately equal length (~10 million year [myr], n = 14) time bins[23,26] (Supplementary Table 2); and (2) stage-level time bins (n = 23) (Supplementary Table 3). The reason for this dual time binning scheme is that the former ensures that time bins sample occurrence data at even time intervals (Jurassic/Cretaceous time bin intervals range from around 2–13 million years), whereas the latter provides a finer scale for investigating changes in diversity. Recent work on Mesozoic tetrapods[6,7,10,27] found that uneven time bin duration might not be problematic in creating spuriously high diversity in longer time bins, and therefore stage level bins are appropriate for use in

diversity studies. A further advantage of using stage level time bins is that they allow for inclusion of a greater number of data points, which is important for increasing the statistical power of our tests involving pairwise comparisons. Each occurrence has an associated stratigraphic range based on the temporal duration of its parent collection, which we use to assign individual minimum and maximum ages. Only occurrences that had their entire stratigraphic range lying within a single time bin were included, in order to avoid over-counting single occurrences in multiple time bins, or incorporation of taxa with high uncertainty in their temporal durations. Although this means that certain geological formations, taxonomic sub-groups, or certain collections are excluded from the binned datasets, such omissions are likely to be randomly distributed and should not affect the overall results of the present study.

Shareholder Quorum Subsampling. The shape of diversity curves is strongly influenced by the uneven distribution of data in each of our bins based on the amount of fossils available to sample[26,28]. Numerous methods have been developed in order to account for this heterogeneity, including model-based and phylogenetic methods. The robustness of modelling approaches, in particular estimates of 'residual' diversity, has recently been questioned, and may not reproduce faithful estimates of diversity[29]. By using collection-based occurrence datasets, this variation in sampling can be compensated for by applying subsampling techniques[28,30]. Shareholder quorum subsampling (SQS) standardises in-bin taxonomic occurrence samples based on an estimate of coverage to determine the relative magnitude of taxonomic biodiversity trends[23,30], and has recently gained popularity in application to tetrapod groups[10-12,31]. One recent study (available as a pre-print) has questioned the applicability of SQS due to its sensitivity to changes in the species-abundance distribution [32]. Other methods such as TRiPS have been recently developed[33], but have not been shown to consistently outperform diversity estimation using SQS as of yet. In the SQS method, each taxon is treated as a 'shareholder', whose 'share' is its relative occurrence frequency[30]. Taxa are randomly drawn from in-bin lists, and when a summed proportion of these 'shares' reaches a certain threshold, or 'quorum' (q), subsampling stops and the number of taxa are tallied. Coverage is defined as the proportion of

the frequency distribution of taxa within a sample, and given by the following equation, where O is the number of individual occurrences, and n_1 is the number of singleton occurrences (i.e., taxa sampled exactly once per bin):

$$u = 1 - n_1/O$$

Coverage is estimated by using randomised subsampling to calculate the mean value of Good's *u*[34], the sum of the frequencies of sampled taxa within an occurrence list, and for each time bin, *u* is divided into *q*[30]. Coverage of zero shows that either there are no sampled occurrences or all taxa are singletons, whereas higher coverage indicates more even sampling of taxa. The major difference between SQS and traditional subsampling methods (e.g., rarefaction) is that instead of using a fixed uniform quota[35], it uses coverage which is flexible in response to the changing taxon occurrence distribution. Singletons were excluded, and dominant taxa (those with the highest frequency of occurrences per bin) were included. For each subsampling trial, this treatment of dominant taxa (o_1) means that when this taxon is encountered its share does not contribute towards *q*, but 1 is added to the subsampled diversity estimate for that bin [30]. Additionally, occurrence lists might be biased by single large collections which can create the artificial appearance of poor coverage[30]. This can be resolved by counting occurrences of taxa that only occur in single publications (p_1) as opposed to those which occur in single collections, and to exclude taxa that are only ever found in the most diverse collection (*t*). Together, this correction for dominant taxa and large collections means that Good's *u* is given by the following modified equation[30]:

$$u_1 = (O - o_1 - p_1 + t)/O - o_1$$

SQS was applied to our occurrence datasets for each higher taxonomic group and for each time interval to provide an estimate of global subsampled taxonomic diversity. By subsampling higher taxonomic groups individually, instead of as a whole, we also alleviate the issue of taxon-

specific factors that influence taxonomic identification of fossils, such as variation in identification difficulty, and degree and mode of preservation[30]. This was conducted for both of our binning strategies (see above). SQS was implemented using a Perl script (version 4.3) provided by J. Alroy. The input for this script requires an occurrence dataset and pre-defined scale defining time bins from which to subsample (see Supplementary Tables 1 and 2, and Supplementary Data 1). We ran 1000 sub-sampling trials for each group, and report the mean diversity. For each sequential subsampling iteration, whenever a collection from a new publication was sampled from the occurrence list, subsequent collections were sampled until exactly three collections from that publication had been selected[30,36]. We set a baseline quorum of 0.4, as this has been demonstrated to be sufficient to accurately assess changes in biodiversity[23], and use the results from these as the basis for modelling our extrinsic parameters (see below). In addition to this, we explored the impact of different quorum levels at increments of 0.1 on resulting diversity curves. Full results are reported in Supplementary Data 3 and Supplementary Data 4. Although it has been suggested that SQS can remove a underlying signal that controls both sampling and biodiversity [37], Mannion *et al.*[10] and Tennant *et al.*[20] found that the relationships between environmental variables and diversity were strengthened, and relationships between taxonomic diversity and sampling proxies were weakened, upon application of SQS, which suggests that signal dampening is unlikely to be a problem.

SQS results with a varying quorum. If we increase the subsampling quorum level (i.e., simulate more even sampling), we can estimate with greater accuracy the magnitude of any diversity decline across the J/K boundary in groups that are relatively well sampled (Supplementary Data 4). Marine crocodyliforms (primarily Thalattosuchia) suffered a maximum J/K boundary decline of 57% at a quorum of 0.6, only slightly higher than using a quorum of 0.4. Non-marine crocodyliforms exhibited a similar level of decline, 60% with a quorum of 0.5, from the Tithonian–Berriasian, slightly higher again than estimates using a quorum of 0.4. The magnitude of the lepidosauromorph radiation becomes dampened to just a 30% increase (quorum of 0.5), and in mammals the Berriasian–Valanginian decline becomes more striking with a 73% drop of diversity (quorum of 0.7). For

dinosaurian faunas across the J/K boundary, the maximum level of decline in ornithischians reached 37% (quorum of 0.8), and 76% in sauropods from the Tithonian–Valanginian (quorum of 0.6). The crash in theropod diversity is greatly emphasised at higher quorum levels, with a maximum estimated loss of 85% at a quorum of 0.7. Pterosaurs remain too poorly sampled throughout the majority of the Early Cretaceous to adequately assess global subsampled diversity patterns, as are turtles at quorum levels higher than 0.4. The magnitude of diversity decline in marine reptiles is also emphasised at higher quorum levels, matching previous estimates using residual diversity[6,7], although the timing is difficult to pinpoint with ichthyosaurs suffering a maximum diversity loss of 71% from the Tithonian–Hauterivian, and sauropterygians losing 74% of their diversity from the Tithonian–Valanginian.

Bootstrapping SQS. In addition to the SQS trials using the Perl script, we also employed a second stream of analyses based on the form of SQS for R (version 3.3). The code for this is freely available from the site of John Alroy (http://bio.mq.edu.au/~jalroy/SQS-3-3.R), and we provide an updated version by, extending the function to have a bootstrapping component (Supplementary Data 9). This version of SQS produces slightly different results to the Perl script, but does not influence the overall shape of diversity, at least for Crocodyliformes[20]. For each clade we performed 1000 bootstrap iterations for SQS, replacing each taxon back in the list after each iteration. We calculated the median (50% confidence interval) and 5% and 95% confidence intervals as lower and upper bounds, respectively (Supplementary Data 3).

Phylogenetic diversity estimation. While phylogenetic methods might be superior for estimation of extinction and origination rates, conducting such analyses for all Tetrapoda is beyond the scope of the present study. Additionally, phylogenetic approaches can have the adverse effect of imposing an asymmetry in analyses by only correcting origination times and not extinction times, and are also highly sensitive to changes in phylogenetic hypotheses and the selective inclusion of only well-

known taxa[38]. However, one of the major advantages of using PDEs is that they generate diversity estimates even in very poorly sampled intervals due to the continuous nature of phylogenetic branches, as opposed to discrete taxonomic occurrences. Although producing comprehensive trees for all J/K tetrapods is beyond the scope of our study, it is possible to produce phylogenetic diversity estimates (PDE) at an individual clade level based on pre-existing data. These independent results can then be compared with those obtained through subsampling.

To calculate PDEs, our source trees were obtained from the primary research literature for the three major clades of dinosaurs[39], sauropterygians[40], ichthyopterygians[41], and pterosaurs[42]. Other tetrapod groups lack well-sampled trees for our study interval. Trees were all time-scaled using the equal method in the R package *strap* [43] using the DatePhylo() function. This works by assigning an equal portion of time to zero-length branches available from the first directly ancestral branch of positive length. Trees were dated using taxonomic first (FAD) and last (LAD) occurrences extracted from the PaleoDB. PDEs were calculated based on the sum of known lineages and implied ghost lineages at the stage level (Supplementary Data 3).

Extinction and origination rates. Extinction and origination rates were calculated for the global occurrence datasets for each higher taxonomic group based on two different measures. We did not calculate regional origination and extinction rates, because at this level it becomes impossible to distinguish between these and other events, including migration and localised extirpations. 'Foote' rates[44,45] are based on cohort analysis (i.e., the number of taxa present in the base of a bin), and is essentially a boundary-crosser method. The equations for calculating extinction (μ) and origination (λ) rates are:

$$\mu = ln(N_b + N_{bt})/N_{bt}$$

$$\lambda = ln(N_t + N_{bt})/N_{bt}$$

Taxa that cross both boundaries are given by N_{bt}, and N_b and N_t indicate taxa that cross the bottom and top boundaries of a bin, respectively. 'The Foote' methods are considered to be conservative estimates of rates, as they assume perfect sampling, and ignore singletons[46], but can also suffer from 'edge' effects and the back-smearing of extinction rates[36]. The advantage of boundary-crossing methods, with respect to in-bin methods, is that the former compensates for potential issues with grouping taxa within bins that might not have temporally co-existed[46].

The second method calculates three-timer (3T) rates[22,36], with three-timers defined as taxa which are found immediately before, within, and immediately after a bin[30]. 3T rates are corrected for the fact that members of this group might be present but not sampled in the following bin (i.e., the Signor-Lipps effect [47]). The equations for extinction (μ_1) and origination (λ_1) rates are:

$$\mu_1 = ln(N_{2t,i}/N_{3t,i}) + \ln(N_{3t}/(N_{3t} + N_{pt}))$$

$$\lambda_1 = ln(N_{2t,i+1}/N_{3t,i}) + \ln(N_{3t}/(N_{3t} + N_{pt}))$$

Here, N_{2t} represents the two-timer cohort, N_{3t} represents the 3T cohort, and the denominator represents the conditional probability of a taxon being sampled given that it is definitely present throughout the time bin [22,30], with N_{pt} being the number of taxa sampled immediately before and after an interval, but not within it (i.e., part-timers). As in previous studies that have used such approaches, we do not assess whether or not the changes in origination and extinction rates are statistically significant, as our analysis trials return only a single raw value.

Addressing the impact of megabiases. We used a range of data series representing different sampling proxies to account for the impact of heterogeneous sampling of the Jurassic/Cretaceous tetrapod fossil record. Sampling proxies have been broadly used to account for some aspect of sampling, including geological and anthropogenic factors, which introduce error into resulting diversity curves[6-9,25,48,49]. This is based on the expectancy that as opportunities to sample fossils increase, one would expect the number of taxonomic units sampled, and hence raw diversity, to

increase proportionally. The use of sampling proxies, therefore, is to alleviate this lack of evenness of sampling through space and time, by either standardising samples or ‘correcting’ raw diversity for sampling bias. Commonly used proxies include the number of fossil-bearing geological formations for the target group[6-8,11,14-16,24,25,50], rock outcrop area or volume[51-55], and the number of fossiliferous collections[15,22,24,26,28,30,56-60]. These proxies represent some factor of sampling bias that accounts for incomplete and heterogeneous spatiotemporal sampling of different palaeoenvironments and the fossil record, including variations in rock volume, accessibility of exposed units, and variations in collecting effort

Geological formations represent the formally recognised units of global stratigraphy, and are defined as lithologically distinct and mappable rock units. The use of formation counts as a proxy for the rock record has been debated in detail recently, in particular with regard to their usage in the “correction” of diversity curves for geological sampling bias[25,48,61-64]. This is due to the fact that they might not be directly comparable units because of inherent features including: (1) variations in thickness; (2) varying preservation potentials for fossils; (3) differences in the time of discovery and the amount of time to accumulate fossils; (4) different naming procedures for geopolitical reasons; (5) the extent to which they are exposed and correlate with outcrop (map) area; (6) different sedimentological and environmental conditions of formation; and (7) different temporal durations. Irrespective of this variation, the fact that numerous studies have found significant correlations with additional, non-redundant proxies for the rock record[50,65] (though see [48,49,66,67]), suggests that fossil-bearing formation counts could represent an adequate proxy for the amount of rock record available for sampling.

In addition to this is the issue of ‘redundancy’, which arises from non-independence of sampling proxies and diversity when calculated at the same taxonomic level[25,61,62,68] (e.g., comparing dinosaur-bearing formations to dinosaur diversity). One method for mitigating these potential issues is to use a sampling proxy that represents a more inclusive clade that includes the target clade[24,27,58,69], e,g, comparing tetrapod-bearing formations to dinosaur diversity. Therefore, for the marine and non-marine realms, we calculated the number of marine (MBF) and non-marine

tetrapod-bearing formations (TBF) at a global level, respectively, and also separately for North America and Europe, and tested their relationships with uncorrected and subsampled diversity for all tetrapod groups. TBFs are defined as any named geological formation that has ever yielded a published tetrapod body fossil, irrespective of the completeness of specimens, based on records within the PaleoDB (Supplementary Data 1). Where both non-marine and marine fossils co-occur within a formation, the formation is added to both marine (MBF) and non-marine (TBF) data series. In addition to accounting for redundancy, the use of a tetrapod-level metric also accounts for failure to sample fossils from individual tetrapod groups, which would be obscured in lower level formation proxies. Furthermore, the use of a tetrapod-level proxy reflects the total potential of the geological record to yield fossils, and overcomes failure to find specimens of individual groups based either on lower abundance of fossil groups, or genuine opportunities to sample them. TBF data series were divided into stage and 10 myr time bins in the same manner as for our occurrence data sets. Note that this means that single formations with long temporal durations can be included in multiple time bins, as long as fossils occur within each bin a formation occupies.

To assess the impact of anthropogenic factors that might bias sampling, we used a metric based on the number of collections in the PaleoDB. Collections-based sampling metrics are representative of irreducible instances in which fossils have been sampled from a particular stratigraphic horizon at a discrete location. They have been commonly used as the basis to reconstruct standardised diversity through sub-sampling approaches[23,30,56]. PaleoDB collections are a proxy for anthropogenic factors, such as sampling of fossil-bearing localities, and worker effort on fossils from those localities reflected in the published literature. They are underpinned by the availability of opportunities to sample from particular rock units, and therefore undoubtedly also capture an aspect of heterogeneity in the availability of fossil-bearing rock units. Correlations between raw diversity and collection counts have also been interpreted as resulting from redundancy rather than representing a valid metric for sampling[25,62]. To account for this we used a 'higher level' metric of tetrapod-bearing formations and collections that should reduce any potential redundancy. We followed the same time series analysis protocol (see Methods section) as for all

other extrinsic factors to test the relationship between collection counts and uncorrected and subsampled diversity.

Outcrop area has been used widely as a metric to quantify the availability of fossiliferous rock from which to sample[51,53,54,67,70], and some other studies have used ‘packages’ as a way of estimating rock record volume[50,65]. The use of outcrop area as a sampling proxy remains questionable due to inconsistency in its relationship with the amount of rock available from which to sample fossils (i.e., exposure area)[66]. Global data of the sedimentary rock record have not been compiled at stratigraphic scales finer than epochs[71], and therefore testing the relationship between rock outcrop area and diversity is limited to a regional scale, which is the preferable approach when using this proxy[66]. Regional records have been compiled for North America, based on sediment coverage area from the COSUNA dataset (Correlation of Stratigraphic Units of North America)[72], and for western Europe using outcrop occurrences for map-based collections[54]. We used the marine and terrestrial units from Peters and Heim[72], and the total rock estimate based on an equal-grid sampling method from Smith and McGowan[54] to represent non-redundant quantifications of the amount of sedimentary rock available for sampling from North America and western Europe, respectively. This aspect of non-redundancy is particularly important here[51,61,62], because macrostratigraphic measures of the rock record are independent of the fossil record[72]. By using both geological and collection-based proxies, we are able to address the two major modes of bias that can be potentially introduced into fossil occurrence datasets.

In addition to the above, we also tested the relationship between global sampling and sea level for evidence of the ‘common cause’ hypothesis, which explains that short-term fluctuations in both sampling and diversity are related to a third, external factor, such as sea level, temperature, or continental weathering rates[37,65,72,73]. While a common cause factor might manifest itself on a smaller-scale regional level[64,74], Butler *et al.*[15] found no significant correlation between short-term (i.e., detrended) fluctuations in global sampling effort or dinosaur diversity and sea level. By using this complete set of proxies, we should be able to distinguish whether relationships between diversity and sampling are correlated through sampling bias, redundancy, or common cause[25,62].

Supplementary References

1 Cohen, K. M., Finney, S. C., Gibbard, P. L. & Fan, J.-X. The ICS International Chronostratigraphic Chart. *International Commission on Stratigraphy* **36** (2013).

2 Vennari, V. V. *et al.* New constraints on the Jurassic–Cretaceous boundary in the High Andes using high-precision U–Pb data. *Gondwana Research* **26**, 374-385 (2014).

3 Mahoney, J., Duncan, R., Tejada, M., Sager, W. & Bralower, T. Jurassic-Cretaceous boundary age and mid-ocean-ridge–type mantle source for Shatsky Rise. *Geology* **33**, 185-188 (2005).

4 Wimbledon, W. *et al.* Fixing a basal Berriasian and Jurassic/Cretaceous (J/K) boundary—is there perhaps some light at the end of the tunnel. *Rivista Italiana di Paleontologia e Stratigrafia* **117**, 295-307 (2011).

5 Carrano, M. T., Alroy, J., Mannion, P. D., Benson, R. & Butler, R. J. Taxonomic occurrences of Jurassic to Cretaceous Tetrapoda recorded in , Fossilworks, the Evolution of Terrestrial Ecosystems database, and the Paleobiology Database, https://paleobiodb.org/. (2015).

6 Benson, R. B. J. & Butler, R. J. in *Comparing the Geological and Fossil Records: Implications for Biodiversity Studies* Vol. 358 (eds A. J. McGowan & A. B. Smith) 191-208 (Geological Society of London, Special Publications, 2011).

7 Benson, R. B. J., Butler, R. J., Lindgren, J. & Smith, A. S. Mesozoic marine tetrapod diversity: mass extinctions and temporal heterogeneity in geological megabiases affecting vertebrates. *Proceedings of the Royal Society Series B: Biological Sciences* **277**, 829-834, doi:DOI 10.1098/rspb.2009.1845 (2010).

8 Benson, R. B. J. *et al.* Cretaceous tetrapod fossil record sampling and faunal turnover: Implications for biogeography and the rise of modern clades. *Palaeogeogr Palaeocl* **372**, 88-107, doi:DOI 10.1016/j.palaeo.2012.10.028 (2013).

9 Butler, R. J., Benson, R. B. J. & Barrett, P. M. Pterosaur diversity: Untangling the influence of sampling biases, Lagerstätten, and genuine biodiversity signals. *Palaeogeogr Palaeocl* **372**, 78-87, doi:DOI 10.1016/j.palaeo.2012.08.012 (2013).

10 Mannion, P. D. *et al.* Climate constrains the evolutionary history and biodiversity of crocodylians. *Nature Communications* **6**, 8438, doi:10.1038/ncomms9438 (2015).

11 Newham, E., Benson, R., Upchurch, P. & Goswami, A. Mesozoic mammaliaform diversity: The effect of sampling corrections on reconstructions of evolutionary dynamics. *Palaeogeography Palaeoclimatology Palaeoecology* **412**, 32-44, doi:DOI 10.1016/j.palaeo.2014.07.017 (2014).

12 Nicholson, D. B., Holroyd, P. A., Benson, R. B. & Barrett, P. M. Climate-mediated diversification of turtles in the Cretaceous. *Nature Communications* **6**, 1-8, doi:10.1038/ncomms8848 (2015).

13 Upchurch, P., Mannion, P., Benson, R., Butler, R. & Carrano, M. Geological and anthropogenic controls on the sampling of the terrestrial fossil record: a case study from the Dinosauria. *Geological Society, London, Special Publications* **358**, 209-240 (2011).

14 Butler, R. J., Barrett, P. M., Nowbath, S. & Upchurch, P. Estimating the effects of sampling biases on pterosaur diversity patterns: implications for hypotheses of bird/pterosaur competitive replacement. *Paleobiology* **35**, 432-446, doi:10.1666/0094-8373-35.3.432 (2009).

15 Butler, R. J., Benson, R. B. J., Carrano, M. T., Mannion, P. D. & Upchurch, P. Sea level, dinosaur diversity and sampling biases: investigating the 'common cause' hypothesis in the terrestrial realm. *Proceedings of the Royal Society of London Series B: Biological Sciences* **278**, 1165-1170, doi:DOI 10.1098/rspb.2010.1754 (2011).

16 Butler, R. J., Brusatte, S. L., Andres, B. & Benson, R. B. How do geological sampling biases affect studies of morphological evolution in deep time? A case study of pterosaur (Reptilia: Archosauria) disparity. *Evolution* **66**, 147-162 (2012).

17 Robeck, H. E., Maley, C. C. & Donoghue, M. J. Taxonomy and temporal diversity patterns. *Paleobiology* **26**, 171-187, doi:Doi 10.1666/0094-8373(2000)026<0171:Tatdp>2.0.Co;2 (2000).

18 Hendricks, J. R., Saupe, E. E., Myers, C. E., Hermsen, E. J. & Allmon, W. D. The Generification of the Fossil Record. *Paleobiology* **40**, 511-528, doi:10.1666/13076 (2014).

19 Barrett, P. M., McGowan, A. J. & Page, V. Dinosaur diversity and the rock record. *Proceedings of the Royal Society Series B: Biological Sciences* **276**, 2667-2674, doi:DOI 10.1098/rspb.2009.0352 (2009).

20 Tennant, J. P., Mannion, P. D. & Upchurch, P. Environmental drivers of crocodyliform extinction across the Jurassic/Cretaceous transition. *Proceedings of the Royal Society of London B: Biological Sciences* **283**, doi:10.1098/rspb.2015.2840 (2016).

21 Gradstein, F. M., Ogg, J. G., Schmitz, M. & Ogg, G. *The Geologic Time Scale 2012*. Vol. 2 (Elsevier, 2012).

22 Alroy, J. Dynamics of origination and extinction in the marine fossil record. *Proceedings of the National Academy of Sciences* **105**, 11536-11542 (2008).

23 Alroy, J. Geographical, environmental and intrinsic biotic controls on Phanerozoic marine diversification. *Palaeontology* **53**, 1211-1235 (2010).

24 Brocklehurst, N., Kammerer, C. F. & Frobisch, J. The early evolution of synapsids, and the influence of sampling on their fossil record. *Paleobiology* **39**, 470-490, doi:Doi 10.1666/12049 (2013).

25 Benton, M. J., Ruta, M., Dunhill, A. M. & Sakamoto, M. The first half of tetrapod evolution, sampling proxies, and fossil record quality. *Palaeogeogr Palaeocl* **372**, 18-41, doi:DOI 10.1016/j.palaeo.2012.09.005 (2013).

26 Alroy, J. *et al.* Phanerozoic trends in the global diversity of marine invertebrates. *Science* **321**, 97-100, doi:DOI 10.1126/science.1156963 (2008).

27 Mannion, P. D., Upchurch, P., Carrano, M. T. & Barrett, P. M. Testing the effect of the rock record on diversity: a multidisciplinary approach to elucidating the generic richness of sauropodomorph dinosaurs through time. *Biological Reviews* **86**, 157-181, doi:DOI 10.1111/j.1469-185X.2010.00139.x (2011).

28 Alroy, J. *et al.* Effects of a sampling standardization on estimates of Phanerozoic marine diversification. *Proceedings of the National Academy of Sciences of North America* **98**, 6261-6266 (2001).

29 Brocklehurst, N. A simulation-based examination of residual diversity estimates as a method of correcting for sampling bias. *Palaeontologia Electronica* **18**, 1-15 (2015).

30 Alroy, J. in *Quantitative Methods in Paleobiology* Vol. 16 (ed G. Hunt J. Alroy) 55-80 (The Paleontological Society, 2010).

31 Mannion, P. D. *et al.* A temperate palaeodiversity peak in Mesozoic dinosaurs and evidence for Late Cretaceous geographical partitioning. *Global Ecology and Biogeography* **21**, 898-908 (2012).

32 Hannisdal, B., Haaga, K. A., Reitan, T., Diego, D. & Liow, L. H. Common species link global ecosystems to climate change. *bioRxiv*, 043729 (2016).

33 Starrfelt, J. & Liow, L. H. How many dinosaur species were there? Fossil bias and true richness estimated using a Poisson sampling model. *Philosophical Transactions of the Royal Society B: Biological Sciences* **371**, 20150219 (2016).

34 Good, I. J. The population frequencies of species and the estimation of population parameters. *Biometrika* **403**, 237-264 (1953).

35 Raup, D. M. Taxonomic diversity estimation using rarefaction. *Paleobiology* **1**, 333-342 (1975).

36 Alroy, J. Accurate and precise estimates of origination and extinction rates. *Paleobiology* **40**, 374-397, doi:Doi 10.1666/13036 (2014).

37 Hannisdal, B. & Peters, S. E. Phanerozoic Earth system evolution and marine biodiversity. *Science* **334**, 1121-1124, doi:DOI 10.1126/science.1210695 (2011).

38 Lane, A., Janis, C. M. & Sepkoski, J. J. Estimating paleodiversities: a test of the taxic and phylogenetic methods. *Paleobiology* **31**, 21-34, doi:Doi 10.1666/0094-8373(2005)031<0021:Epatot>2.0.Co;2 (2005).

39 Sakamoto, M., Benton, M. J. & Venditti, C. Dinosaurs in decline tens of millions of years before their final extinction. *Proceedings of the National Academy of Sciences of the United States of America* **113**, 5036-5040, doi:10.1073/pnas.1521478113 (2016).

40 Fischer, V. *et al.* Peculiar macrophagous adaptations in a new Cretaceous pliosaurid. *Royal Society Open Science* **2**, doi:10.1098/rsos.150552 (2015).

41 Fischer, V. *et al.* New ophthalmosaurid ichthyosaurs from the European Lower Cretaceous demonstrate extensive ichthyosaur survival across the Jurassic-Cretaceous boundary. *PLOS ONE* **7**, e29234, doi:10.1371/journal.pone.0029234 (2012).

42 Upchurch, P., Andres, B., Butler, R. J. & Barrett, P. M. An analysis of pterosaurian biogeography: implications for the evolutionary history and fossil record quality of the first flying vertebrates. *Historical Biology* **27**, 697-717 (2014).

43 Bell, M. A. & Lloyd, G. T. strap: an R package for plotting phylogenies against stratigraphy and assessing their stratigraphic congruence. *Palaeontology* **58**, 379-389 (2015).

44 Foote, M. Origination and extinction components of taxonomic diversity: general problems. *Paleobiology* **26**, 74-102 (2000).

45 Foote, M. Origination and extinction through the Phanerozoic: a new approach. *The Journal of Geology* **111**, 125-148 (2003).

46 Foote, M. Origination and extinction through the Phanerozoic: A new approach. *J Geol* **111**, 125-148, doi:Doi 10.1086/345841 (2003).

47 Signor, P. W. & Lipps, L. H. Sampling bias, gradual extinction patterns and catastrophes in the fossil record. *Geological Society of America Special Papers* **190**, 351-367 (1982).

48 Dunhill, A. M., Benton, M. J., Newell, A. J. & Twitchett, R. J. Completeness of the fossil record and the validity of sampling proxies: a case study from the Triassic of England and Wales. *Journal of the Geological Society* **170**, 291-300, doi:Doi 10.1144/Jgs2012-025 (2013).

49 Dunhill, A. M., Benton, M. J., Twitchett, R. J. & Newell, A. J. Completeness of the Fossil Record and the Validity of Sampling Proxies at Outcrop Level. *Palaeontology* **55**, 1155-1175, doi:DOI 10.1111/j.1475-4983.2012.01149.x (2012).

50 Peters, S. E. & Foote, M. Biodiversity in the Phanerozoic: a reinterpretation. *Paleobiology* **27**, 583-601, doi:Doi 10.1666/0094-8373(2001)027<0583:Bitpar>2.0.Co;2 (2001).

51 Smith, A. B. Marine diversity through the Phanerozoic: problems and prospects. *Journal of the Geological Society* **164**, 731-745, doi:Doi 10.1144/0016/76492006-184 (2007).

52 Smith, A. B. & Benson, R. B. J. Marine diversity in the geological record and its relationship to surviving bedrock area, lithofacies diversity, and original marine shelf area. *Geology* **41**, 171-174, doi:Doi 10.1130/G33773.1 (2013).

53 Smith, A. B., Lloyd, G. T. & McGowan, A. J. Phanerozoic marine diversity: rock record modelling provides an independent test of large-scale trends. *Proceedings of the Royal Society of London Series B: Biological Sciences* **279**, 4489-4495, doi:DOI 10.1098/rspb.2012.1793 (2012).

54 Smith, A. B. & McGowan, A. J. The shape of the Phanerozoic marine palaeodiversity curve: How much can be predicted from the sedimentary rock record of western Europe? *Palaeontology* **50**, 765-774, doi:DOI 10.1111/j.1475-4983.2007.00693.x (2007).

55 Wall, P. D., Ivany, L. C. & Wilkinson, B. H. in *Comparing the Geological and Fossil Records: Implications for Biodiversity Studies* (eds A. B. Smith & A. J. McGowan) 43-62 (Geological Society of London, 2011).

56 Alroy, J. The shifting balance of diversity among major marine animal groups. *Science* **329**, 1191-1194 (2010).

57 Brusatte, S. L., Benton, M. J., Lloyd, G. T., Ruta, M. & Wang, S. C. Macroevolutionary patterns in the evolutionary radiation of archosaurs (Tetrapoda: Diapsida). *Earth and Environmental Science Transactions of the Royal Society of Edinburgh* **101**, 367-382, doi:Doi 10.1017/S1755691011020056 (2010).

58 Cleary, T. J., Moon, B. C., Dunhill, A. M. & Benton, M. J. The fossil record of ichthyosaurs, completeness metrics and sampling biases. *Palaeontology* **58**, 521-536 (2015).

59 Fröbisch, J. Vertebrate diversity across the end-Permian mass extinction—Separating biological and geological signals. *Palaeogeography, Palaeoclimatology, Palaeoecology* **372**, 50-61 (2013).

60 Mannion, P. D. & Upchurch, P. A re-evaluation of the 'mid-Cretaceous sauropod hiatus' and the impact of uneven sampling of the fossil record on patterns of regional dinosaur extinction. *Palaeogeography, Palaeoclimatology, Palaeoecology* **299**, 529-540 (2011).

61 Benton, M. J. Palaeodiversity and formation counts: redundancy or bias? *Palaeontology* **58**, 1003-1029 (2015).

62 Benton, M. J., Dunhill, A. M., Lloyd, G. T. & Marx, F. G. Assessing the quality of the fossil record: insights from vertebrates. *Geological Society, London, Special Publications* **358**, 63-94 (2011).

63 Dunhill, A. M., Benton, M. J., Twitchett, R. J. & Newell, A. J. Completeness of the fossil record and the validity of sampling proxies at outcrop level. *Palaeontology* **55**, 1155-1175 (2012).

64 Dunhill, A. M., Benton, M. J., Twitchett, R. J. & Newell, A. J. Testing the fossil record: Sampling proxies and scaling in the British Triassic–Jurassic. *Palaeogeography, Palaeoclimatology, Palaeoecology* **404**, 1-11 (2014).

65 Peters, S. E. Geologic constraints on the macroevolutionary history of marine animals. *Proceedings of the National Academy of Sciences* **102**, 12326-12331, doi:DOI 10.1073/pnas.0502616102 (2005).

66 Dunhill, A. M. Problems with using rock outcrop area as a paleontological sampling proxy: rock outcrop and exposure area compared with coastal proximity, topography, land use, and lithology. *Paleobiology* **38**, 126-143, doi:Doi 10.1666/10062.1 (2012).

67 Crampton, J. S. *et al.* Estimating the rock volume bias in paleobiodiversity studies. *Science* **301**, 358-360 (2003).

68 Dunhill, A. M., Hannisdal, B. & Benton, M. J. Disentangling rock record bias and common-cause from redundancy in the British fossil record. *Nature Communications* **5**, 4818 (2014).

69 Upchurch, P. & Barrett, P. M. in *The Sauropods: Evolution and Paleobiology* (eds K. A. Curry Rogers & J. A. Wilson) 104-124 (University of California Press, 2005).

70 McGowan, A. J. & Smith, A. B. Are global Phanerozoic marine diversity curves truly global? A study of the relationship between regional rock records and global Phanerozoic marine diversity. *Paleobiology* **34**, 80-103, doi:Doi 10.1666/07019.1 (2008).

71 Wall, P. D., Ivany, L. C. & Wilkinson, B. H. Revisiting Raup: exploring the influence of outcrop area on diversity in light of modern sample-standardization techniques. *Paleobiology* **35**, 146-167 (2009).

72 Peters, S. E. & Heim, N. A. The geological completeness of paleontological sampling in North America. *Paleobiology* **36**, 61-79 (2010).

73 Sepkoski Jr, J. J., Bambach, R. K., Raup, D. M. & Valentine, J. W. Phanerozoic marine diversity and the fossil record. *Nature* **293**, 435-437 (1981).

74 Dunhill, A. M. Using remote sensing and a geographic information system to quantify rock exposure area in England and Wales: Implications for paleodiversity studies. *Geology* **39**, 111-114, doi:Doi 10.1130/G31503.1 (2011).
